# UMAOH Calcium Phosphate Coatings Designed for Drug Delivery: Vancomycin, 5-Fluorouracil, Interferon α-2b Case

**DOI:** 10.3390/ma15134643

**Published:** 2022-07-01

**Authors:** Konstantin A. Prosolov, Ekaterina G. Komarova, Ekaterina A. Kazantseva, Aleksandr S. Lozhkomoev, Sergei O. Kazantsev, Olga V. Bakina, Marina V. Mishina, Anastasia P. Zima, Sergei V. Krivoshchekov, Igor A. Khlusov, Yurii P. Sharkeev

**Affiliations:** 1 Laboratory of Physics of Nanostructured Biocomposites, Institute of Strength Physics and Materials Science, Siberian Branch of Russian Academy of Sciences, 634055 Tomsk, Russia; konstprosolov@gmail.com (K.A.P.); kazantseva.ea@ispms.ru (E.A.K.); asl@ispms.ru (A.S.L.); khlusov63@mail.ru (I.A.K.); sharkeev@ispms.ru (Y.P.S.); 2 Department of Strength and Design, National Research Tomsk State University, 634030 Tomsk, Russia; 3 Laboratory of Nanobioengineering, Institute of Strength Physics and Materials Science, Siberian Branch of Russian Academy of Sciences, 634055 Tomsk, Russia; kzso@ispms.ru (S.O.K.); ovbakina@ispms.ru (O.V.B.); 4 Central Research Laboratory, Siberian State Medical University, 634050 Tomsk, Russia; lab.cni@ssmu.ru; 5 Department of Pathophysiology, Siberian State Medical University, 634050 Tomsk, Russia; zima.ap@ssmu.ru; 6 Department of Pharmaceutical Analysis, Siberian State Medical University, 634050 Tomsk, Russia; ksv_tsu@mail.ru; 7 Department of Morphology and General Pathology, Siberian State Medical University, 634050 Tomsk, Russia

**Keywords:** ultrasound-assisted micro-arc oxidation (UMAO), calcium phosphate coating, 5-fluorouracil, interferon α-2b, vancomycin, drug delivery

## Abstract

Drug delivery systems based on calcium phosphate (CaP) coatings have been recently recognized as beneficial drug delivery systems in complex cases of bone diseases for admission of drugs in the localized area, simultaneously inducing osteoinduction because of the bioavailable Ca and P ions. However, micro-arc oxidation (MAO) deposition of CaP does not allow for the formation of a coating with sufficient interconnected porosity for drug delivery purposes. Here, we report on the method to deposit CaP-based coatings using a new hybrid ultrasound-assisted MAO (UMAOH) method for deposition of coatings for drug delivery that could carry various types of drugs, such as cytostatic, antibacterial, or immunomodulatory compositions. Application of UMAOH resulted in coatings with an Ra roughness equal to 3.5 µm, a thickness of 50–55 µm, and a combination of high values of internal and surface porosity, 39 and 28%, respectively. The coating is represented by the monetite phase that is distributed in the matrix of amorphous CaP. Optimal conditions of coating deposition have been determined and used for drug delivery by impregnation with Vancomycin, 5-Fluorouracil, and Interferon-α-2b. Cytotoxicity and antimicrobial activity of the manufactured drug-carrying coatings have been studied using the three different cell lines and methicillin-resistant *S. aureus*.

## 1. Introduction

Drug delivery systems and implants based on that technology are successful for the local treatment of many chronic conditions and diseases [1]. Conventional treatment of bone disease, for example, is usually connected with systematic supplemental treatment with drugs, which could lead to some unwanted side effects such as systemic toxicity, short-term benefits, and patient inconvenience [2]. Implants could provide localized therapy by carrying the needed drug to the implantation site and delivering it promptly, devoid of these problems. However, there is a constant need for improvement in terms of more controlled drug release and precise admission of high local drug concentrations. The problem of pulse or burst release of drugs is a major obstacle for drug delivery systems and drug-loaded implants [3]. To address this problem, many efforts have been made including surface engineering of implants by anodization [4], nanotube formation [5], coating strategies [6,7], micro- and nanoparticles [8,9], and polymerization [10].

From the point of perspective of new materials for drug delivery systems, calcium phosphate (CaP) based materials are amongst the most interesting [8,9]. This material is already widely applied in clinical practice, starting from the granules made of CaP for the treatment of large bone defects [11], to cement [12], to the CaP-based coatings that increase the biocompatibility of the metallic implants and provide vital elements for bone remodeling. There are attempts to manufacture CaP cement or coatings doped with ions of different metals, which could bring new properties to the material, such as antibacterial [13,14], osteogenic [15,16], or angiogenic [17]. However, drug delivery by means of CaP is still a viable and promising approach.

In general, a potential substrate to be used as a drug carrier must have the ability to incorporate a drug, retain it at a specific target site, and deliver it progressively with time in the surrounding tissues [18]. The inherent porosity of CaP material and the possibility to incorporate a drug not only on the surface of the material but in bulk volume that is also dependent on the phase composition of CaP make this material interesting for further study. More than that, upon degradation, a CaP coating does not produce any potentially harmful residues in a way some polymers do [19]. The degradation rate of the CaP coating can be precisely controlled by tailoring the phase composition and, overall, a Ca/P ratio of the material. Thus, phase composition should be modified in such a way that the kinetics of biodegradation are consistent with the rate of bone formation and release of immobilized drug [20]. It should be also noted that amorphous CaP is expected to degrade at a higher rate due to the absence of long-range order bonding to the atomic structure. Hence, it is expected to be easier to detach the atomic layer by consuming less thermodynamic energy [21]. However, the dissolution of the CaP is not only governed by intrinsic properties of the material but is also due to active degradation, which is a result of the cellular activity (i.e., osteoclasts, giant cells, macrophages) [22]. Active degradation of CaPs is mainly mediated by giant cells and osteoclasts, but macrophages are also involved in the phagocytosis of the fragmented particles of the CaP. It was also reported that osteoclasts are responsible for active biodegradation by an act of mediation of local pH that increases the degradation of CaP [23]. The dissolution of CaP is accelerated at low pH media, which are also typically found in endolysosomes and in the vicinity of tumors, providing an advantage in the delivery of drugs into malign zones or cell organelles [9]. Here, it could be concluded that the CaP material is a prospective candidate for drug delivery applications for the treatment of bone tissue defects, including those complicated by pathological diseases (bone infection, tumor, osteoporosis, etc.), as it simultaneously provides bioavailable Ca and P for bone remodeling and could be tailored to prolonged drug release.

Many kinds of research on the use of both commercial and experimental CaP cements as drug carriers have been published in recent years [24,25]. Antibiotics have received a lot of attention because of their wide range of applications: as prophylactics to avoid infections caused by surgical treatments or in the treatment of bone infections, in general. However, anti-inflammatory and anti-cancer drugs have been the center of attention.

For example, 5-Fluorouracil (5-FU) is an antimetabolite drug that has been widely used for the treatment of different types of cancer and, nowadays, as a component in a complex of drugs during chemotherapy [26]. A pyrimidine analog, 5-FU has been used as an antineoplastic agent to treat multiple solid tumors. While 5-FU has predominantly been used for the treatment of colon and breast cancer, recent studies showed that 5-FU can be active against oral squamous cell carcinoma [27]. From that perspective, the use of 5-FU in combination with CaP based drug delivery system could be of interest to fight oral cancer in general dental practice.

Intravenously administered antibiotics, such as vancomycin (VMN), have poor tissue transferability depending on the site of infection, which decreases their therapeutic potential. Therefore, it has been proposed to use CaP-based cement impregnated in VMN [28]. It was described in the research article that VMN released from the CaP during in vivo testing was much higher than the minimum inhibitory concentration and, therefore, this combination is a promising method for the treatment of established postoperative infections in clinical settings.

Interferons (IFN) are a naturally secreted protein family of cytokines that play key roles in mediating antiviral and antigrowth responses and in modulating immune functions [29]. It is rather difficult to introduce IFN to a patient other than in a needle-based approach because the protein has a large size and is disassociated from the gastrointestinal environment. For successful admission, IFN usually requires some carrier, for example, hydrogel [30].

There is a still major challenge to manufacturing an efficient drug carrier system, especially for bone treatment applications. Here, surface engineering of existing metallic implants plays a vital role. As was mentioned above, an electrochemical family of methods for metallic implant surface engineering is considered one of the most promising approaches to manufacturing drug delivery systems. The micro-arc oxidation (MAO) method, also known as plasma electrolytic oxidation (PEO), is a complex plasma–chemical and electrochemical method for the surface modification of medical implants. The MAO makes it possible to create strong, thick, and uniform CaP coatings with high adhesion to the metallic substrates having complex shapes. The process of MAO is a high-temperature electrochemical reaction that undergoes micro-arc discharges on the surface of a metallic substrate placed in an electrolyte. Although the temperature in local plasma micro arcs on the substrates’ surface can increase rather substantially, the structure of the metallic substrate is not usually affected. The resulting coating is usually characterized by increased thickness (up to 400 µm), high coatings’ hardness, strength, low chemical activity, and electrical conductivity [31,32,33]. To vary the properties of MAO coatings, the composition of the electrolyte, elemental composition of the substrate, and electrophysical modes of the deposition process can be changed. To a large extent, the properties of coatings are determined by the parameters of micro-arc discharges, such as their duration, current strength, or electrolyte temperature. 

Recently, a modified MAO method where, during the micro-arc discharges, ultrasonic (US) vibrations in an electrolyte are introduced, has been extensively researched. The modified method makes use of modulation in fluctuations, a chaotic change in electric potentials, currents, and charges during the MAO process [34,35]. When the US propagates through the volume of electrolytes, the areas of rarefaction and compression appear, leading to cavitation. Cavitation phenomena cause a local significant increase in temperatures and pressures and create mechanical stresses at the phase boundaries. Electric discharges arising in cavitation bubbles can also have a significant effect on the processes occurring in the electrolyte medium. According to the [34,35,36], it was reported that the superposition of a US field during MAO is accompanied by cavitation on the electrode surfaces, acceleration of heat and mass transfer in the electrolyte solution, intensification of electrolyte penetration into micropores and cracks, dispersion of particles and liquid electrolyte clusters, degassing, and activation of crystallization and electrochemical processes. High US frequencies at low amplitude create an acoustic field with a high energy level, which makes it possible to intensify the processes of mass transfer in the electrolyte, increase the growth rate of coatings, and also control the composition, structure, and porosity of the deposited coatings [36,37,38]. 

An increase in the intensity of the micro-arc discharges due to the US influence results in both increases in thickness and volume porosity. As was mentioned above, sufficient porosity is one of the main requirements for a successful drug delivery system. On the other hand, an increase in thickness and porosity can lead to a notable decrease in coating to substrate adhesion. Therefore, the use of combined US and MAO deposition should be optimized to manufacture the coating with a required set of properties for the drug delivery system. Due to that, we propose a two-staged hybrid UMAO (UMAOH) deposition that would combine the benefits of conventional MAO and US modified MAO (UMAO) methods for drug delivery applications.

To the best of our knowledge, CaP coatings deposited using UMAOH process that were designed for the drug delivery application have not been yet reported. The aim of the present work is to show the manufacturing route of MAO, UMAO, and new UMAOH coatings followed by a selection of the optimized deposition parameters and coating properties in terms of their physical and mechanical characteristics for subsequent drug immobilization. Drug loading efficiency, cytocompatibility, and antibacterial properties of drug-carrying UMAOH CaP coatings are also described in detail.

## 2. Materials and Methods

### 2.1. Pre- and Treatment Procedures

Commercially pure Ti (ASTM Grade 2) with a thickness of 1 mm and width and length of 10 mm (VSMPO-AVISMA Corp., Verkhnaya Salda, Russia) was used as substrates for deposition. Before the deposition step, Ti samples were mechanically grinded and polished using silicon carbide paper with a grade ranging from P600 to P2000. A US cleaning (Elmasonic S, Elma Schmidbauer GmbH, Singen, Germany) in distilled water and ethanol for 10 min was performed. After that, the samples were dried in the air. 

In order to deposit CaP coatings for drug loading, a “Microarc-3.0” installation (ISPMS SB RAS, Tomsk, Russia) consisting of a pulsed DC power supply, a sample holder which is an electrode (anode), a titanium electrolytic bath as a counter electrode (cathode), US devices, and a computer for process control was used. The electrolyte contained 5 wt.% nanosized HA (Ca_10_(PO_4_)_6_(OH)_2_), 7 wt.% CaCO_3_, 27 wt.% H_3_PO_4_, and distilled water as a balance [39,40]. Stoichiometric HA powder consisting of aggregates of round-shaped nanocrystallites with a size less than 50 nm was synthesized by the mechanochemical method [41,42]. An acidic suspension electrolyte (pH = 1.2–1.5), incorporating an undissolved nano-dispersed HA, was obtained. 

The UMAO process was carried out in an anodic potentiostat regime with the following parameters: MAO pulse frequency of 50 Hz; MAO pulse duration of 100 µs; time of 10 min; anodic voltage of 200 V; sinewave US frequency of 35 kHz; capacity of 100 W. According to our previous results, three deposition regimes were chosen [36,37,38]: I—MAO (control regime without induced US field); II—UMAO (MAO with applied US field); III—UMAOH (hybrid regime consisting of MAO with US influence for 8 min and subsequent MAO without US for 2 min).

### 2.2. Experimental Techniques

Coating structure and morphology were analyzed by scanning electron microscopy (SEM, LEO EVO 50, Carl Zeiss, Oberkochen, Stuttgart, Germany). The thickness was measured using SEM images of the coating cross-sections according to the ASTM E1382-9 standard protocol. The porosity was determined by the metallographic method as the ratio between the total area of pores to the total area of the SEM image, according to the following formula: *P*(%) = Σ*s/*Σ*S_total_* × 100%, where *s*—area occupied by pores, *S_total_*—total area of SEM image. 

Elemental composition was examined using electron dispersive X-ray spectroscopy (EDX) on an INCA system (Oxford Instruments, High Wycombe, UK) coupled to SEM. Experimental equipment was provided by the “Nanotech” Common Center for Collective Use (ISPMS SB RAS, Tomsk, Russia). For the coating characterization, Fourier transformed infrared spectroscopy (FT-IRS) was performed using Nikolet 5700 IR-spectrometer (Thermo Electron Corporation, Madison, WI, USA) in the reflection mode in the wavenumber range of 700–4000 cm^−1^. The pore size distribution and specific surface areas of the coating were obtained by nitrogen adsorption on a Sorbtometr-M (Katakon, Russia) analyzer. Calculations were performed based on the Brunauer–Emmett–Teller (BET) equation. For UMAOH coatings, the specific surface area was found to be equal to 2 m^2^/g.

The phase composition was identified by X-ray diffraction (XRD) in Bragg–Brentano geometry using a Shimadzu XRD-6000 diffractometer. XRD measurements were collected with Cu Kα irradiation, operating at 40 kV and 30 mA in a 2*θ* range between 10° and 80°, with a copper monochromator coupled scan speed of 2.0°/min. For the crystalline phase indentation, the International Centre for Diffraction Data (ICDD) database was used as a reference. The equipment was provided by the Tomsk Materials Science Centre for Collective Use (National Research Tomsk State University, Tomsk, Russia).

### 2.3. Drugs’ Loading and Desorption Studies

In order to carry out the drug’s immobilization into the UMAOH CaP coatings, three groups of drug solution series were prepared by subsequent dilution: VMN (Elfa Laboratories, Russia) of 10, 25, and 50 mg/mL; 5-FU (Lens-Pharm, Russia) of 10, 25, and 50 mg/mL; IFNα-2b (Reaferon EC 5 × 10^6^ IU, Vektor-Medika, Russia) of 0.1 × 10^6^, 0.5 × 10^6^, and 1 × 10^6^ IU/mL. The UMAOH CaP coated samples were soaked in 1 mL of drug solutions at each of the abovementioned concentrations for 1 h at 25 °C. The concentration of drugs in the solution was determined by spectrophotometry using UV/Vis Spectrophotometer SF-2000 (OKB Spectr, Russia) in the wavelength range λ = 200–900 nm. After the soaking, the samples were dried in air at 40 °C for 6 h.

**Figure 1:**
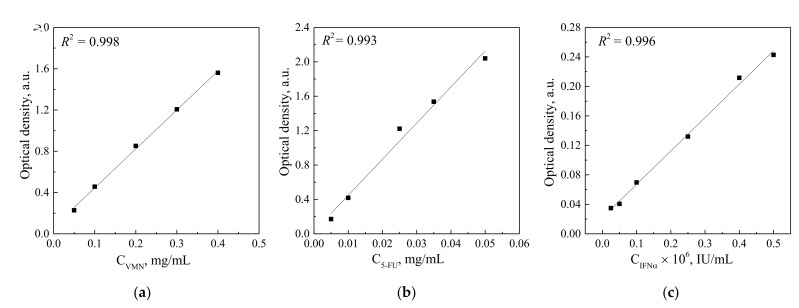
Calibration plot of the optical density against the different drug concentrations in case of VMN (**a**), 5-FU (**b**), and IFNα (**c**).

To study the drug release from the UMAOH CaP coatings, the samples were immersed in separate vials containing 10 mL of 0.9 wt.% NaCl solution in air at 25 °C. The solutions after the drug release were studied by spectrophotometry in predefined time periods up to 120 h. The concentration of the released drug was determined from pre-built calibration curves according to the intensity of the peaks that are characteristic of each drug. (VMN—280 nm, 5-FU—265 nm, IFNα—280 nm.) As a comparison solution, 0.9 wt.% NaCl solution was used (Figure 1). A detailed description of adsorption–desorption experiments is reported in our previous work [43].

#### 2.3.1. Concentration of IFNα in Commercially Available Drug Reaferon EC

High-Performance Liquid Chromatography (HPLC) was used with the help of an Ultimate3000 device (Thermo Fisher Scientific, Munich, Germany). A portion (5.4 mg) of the commercially available Drug Reaferon EC 5 × 10^6^ IU lyophilizate was dissolved in 540 µL of the mobile phase (10% B), centrifuged at 15,000 rpm for 5 min, and analyzed as previously described in [44] under the conditions listed below. Stationary phase: Pinnacle II C18 150 × 4.6 mm, 5 µm, 140 A, column oven temperature 30 °C. Mobile phase (A)—0.1% trifluoroacetic acid in water (Type I); mobile phase (B)—0.1% trifluoroacetic acid in acetonitrile (HPLC grade, Panreac). Gradient mode 0-1 min isocratic 10% B; 1–20 min up to 60% B; 20–25 min isocratic 60% B; 25–35 min—column regeneration 10% B. Detection was at 220 nm. The calculated concentration of IFNα substance in this lyophilizate (using an area normalization method) was 1.2 µg/mg of the officinal drug (23.16 µg of IFNα per ampule).

#### 2.3.2. Enzyme-Linked Immunosorbent Assay of IFNα Releasing UMAOH CaP Coatings Delivery System

To estimate in vitro release of IFNα from the UMAOH CaP coatings, the enzyme-linked immunosorbent assay (ELISA) was carried out using an Alpha-INTERFERON- IFA-BEST kit (Vektor Best, Novosibirsk, Russia), according to the manufacturer protocol, using a Multiskan FC photometer (Termo Fisher Scientific, Waltham, MA, USA). Before the study, UMAOH CaP coatings were immersed in 1 mL of IFNα solution with a concentration of 1 × 10^6^ IU/mL. For the drug release analysis, drug carrying UMAOH CaP coatings were immersed in separate vials for pre-defined time periods (1, 6, 12, 24, and 48 h) containing 10 mL of 0.9 wt.% NaCl solution and stored in air at 25 °C. The solutions after the drug release were studied by ELISA and percent of release was estimated by comparison of obtained results with the initial adsorbed IFNα content per sample, which was estimated based on HPLC and UV−vis data. 

### 2.4. Cytotoxicity In Vitro

Cytotoxicity of UMAOH CaP coatings containing VMN, 5-FU, or IFNα was studied using the following cell lines: 3T3 (mouse non-tumorous embryonic fibroblasts); HeLa (epithelioid cervix carcinoma); MCF-7 (metastatic adenocarcinoma of breast tissue). These well researched and established non-tumorous and tumorous cell lines are frequently used in studies related to cytotoxic activity of new materials [45]. The viability of the cells was determined using standard protocol for MTT-test (ISO 10993-5, ISO 10993-12). Yellow water-soluble MTT (3-(4,5-dimethylthiazol-2-yl)-2,5-diphenyltetrazolium bromide) is metabolically reduced in viable cells to a blue-violet insoluble formazan. The number of viable cells correlates to the color intensity determined by photometric measurements after dissolving the formazan in alcohol. Optical density was determined using a Multiskan FC photometer (Termo Fisher Scientific, Waltham, MA, USA) at 570 nm wavelength.

Before the cytotoxicity test, the UMAOH CaP-coated samples were separated into three groups. The first group of drugs carrying UMAOH CaP coatings was immersed in 5 mL of VMN of 50 mg/mL concentration, the second group was loaded by immersion in 5-FU 5 mL of a solution of 50 mg/mL concentration, and the third group was immersed in IFNα of 1 × 10^6^ IU/mL concentration.

For the cytotoxicity tests, the cells were transferred to a 24-well plate. The cell suspension containing 1 × 10^5^ cells was seeded in the center of each sample and kept in the incubator for 3–5 min to ensure cell adhesion. After that, 1 mL of medium was added to each well. As a positive control in the MTT assay, a drug concentration in 1 mL of medium in each well was as follows: 0.05 mg/mL of VMN, 0.05 mg/mL of 5-FU, and 1 × 10^6^ IU/mL of IFN. The selected concentrations were chosen in order to exceed the maximum possible concentrations of drugs that could be extracted from the samples in the conditions described in Section 2.3. Drug Loading and Desorption Studies. Cells that were incubated on the surface of as-deposited UMAOH CaP samples under the same experimental conditions served as a negative control.

All above-mentioned cell lines were cultured in DMEM-F-12 solution including 10% of Fetal bovine serum (FBS) and 5% streptomycin. For the cultivation of cells, a Sanyo MCO-5AC (Sanyo, Japan) incubator was used, working at the constant temperature of 37 °C and air atmosphere containing 5% CO_2_.

### 2.5. Antibacterial Assay

The antibacterial activity of extracts prepared from UMAOH CaP coatings containing VMN was determined by Agar Diffusion Assays (Kirby–Bauer method) [46] using a methicillin-resistant strain of *S. aureus* (MRSA ATCC4300). 

To obtain an extract from UMAOH CaP coatings containing VMN at concentrations of 10, 25, and 50 μg/mL, the samples were placed in a sterile 0.9% NaCl solution with a volume of 10 mL and kept for 1 h, 6 h, 24 h, 48 h, 96 h, 120 h, and up to 7 days. For each timepoint, separate vials containing the NaCl solution and a sample were used. A total of 72 samples was used in the study. VMN at a concentration of 20 μg/mL was used as a positive control. As a negative control, as-deposited UMAOH CaP coatings were used.

A bacterial suspension of MRSA with a concentration of 10^7^ CFU/mL in 0.9% NaCl solution was placed into nutrient agar medium in 90 mm plastic Petri dishes and spread across the surface with a swab. The Petri dishes were divided into five equal sectors where five sample carriers Rotilabo^®^ (Ø = 6 mm, thickness = 0.75 mm; Carl Roth, Karlsruhe, Germany) were placed. The sample carriers were impregnated with 20 µL of extract obtained from the VMN-containing UMAOH CaP sample. In order to let the drug diffuse from the sample carrier, the Petri dishes were incubated for 24 h at 37 °C and 100% humidity. After the incubation, the diameter of the inhibition zone around the sample carriers, where MRSA growth inhibition occurred, was determined.

According to ISO 20645-2014, the width of the bacterial growth inhibition zone (*H*) was calculated using the formula:

(1) $$H=\frac{D-d}{2}$$

where *D* is the diameter of the inhibition zone around the sample carriers, mm; *d* is the diameter of the sample carrier, mm.

### 2.6. Statistical Analysis

Statistical analysis was carried out using the Origin Pro 8.1 software. The normality of the experimental data distribution was defined by the Kolmogorov–Smirnov test with Lilliefors correction. When describing the normal distribution, the mean (X) ± standard deviation (SD) was used to determine the central tendency. The median (Me) and quartile range (Q_1_–Q_3_) were used for non-normal distribution. A non-parametric Mann–Whitney U-test and Wilcoxon signed-rank test were used to evaluate the significant differences between the independent and dependent groups, respectively. A statistically significant difference was considered at a value of p < 0.05.

## 3. Results and Discussion

### 3.1. Production of UMAO Coatings and Their Characterization

**Figure 2:**
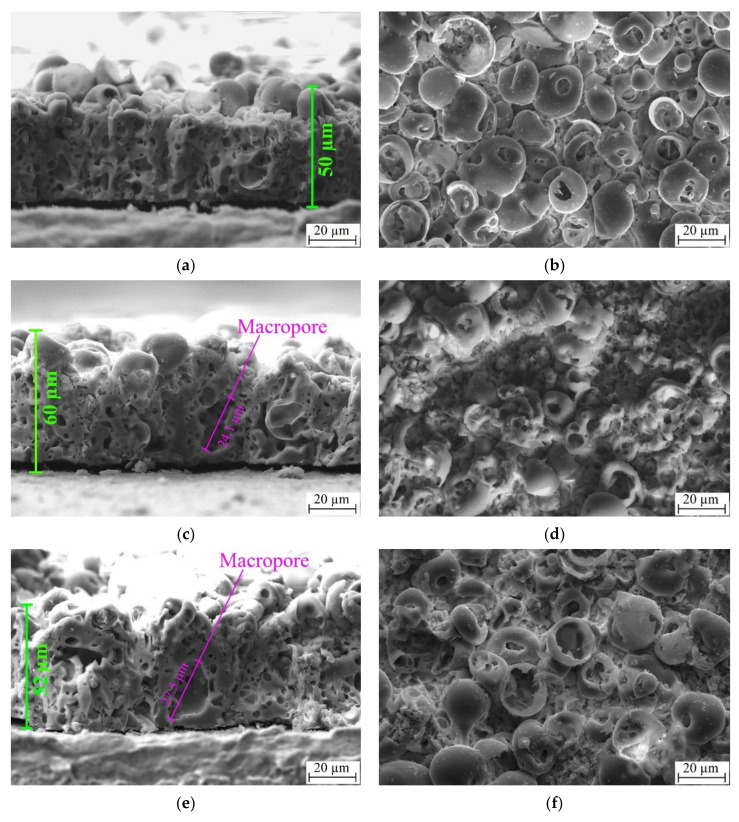
SEM images of the cross-section (**a**, **c**, **e**) and the surface morphology (**b**, **d**, **f**) of the different coatings: MAO coating (**a**, **b**); UMAO coating (**c**, **d**); UMAOH coating (**e**, **f**).

According to the SEM results in Figure 2a,c,e, the deposited coatings are represented by a complex hierarchical structure. The internal structure of the coating is comprised of a system of interconnected pores. The porous structure is represented by channels that extend from the substrate to the coatings’ surface. The coatings’ surface morphology is represented by spheroid-like structural elements (spheres) having pores, not only in their volume, but which are also located in between spheres (Figure 2b,d,f).

**Figure 3:**
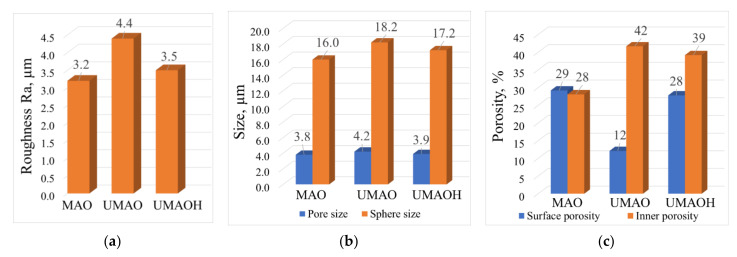
The surface roughness (**a**), the average size of structural elements (**b**), and the surface and inner porosities (**c**) of the MAO, UMAO, and UMAOH coatings.

The SEM of the coatings’ cross-section (Figure 2a,c,e) reveals the influence of the US field, which resulted in a slight increase in thickness from 50 to 60 µm and a significant increase in the average pore size from 2.5 to 4.7 µm. These results represent the following previously published data [38]. The observed results are due to the formation of local macropores having a size in the range of 15–35 µm (Figure 2c,e). At the same time, the influence of the US field leads to a notable morphology change in the coatings’ surface, which is represented in Figure 2b,d,f. In the case of UMAO deposition, areas of distorted or destructed spheres alongside the spheres that are usually observed for conventional surface morphology of MAO deposited coatings can be noted. The residues of destructed spheres led to a notable morphology difference that is due to the decrease in the number of voids in between pores (Figure 2d). In contrast, the coatings deposited using UMAOH mode are characterized by a localized area of destructed spheres. This is because, during the last 2 min of deposition when the US field is no longer present, the spheres that are characteristic of a conventional MAO mode are deposited on the surface (Figure 2e). The observed morphological changes for the coatings deposited using UMAO mode led to a slight increase in surface roughness, from 3.2 to 4.4 µm (Figure 3a), while the UMAOH coatings’ roughness is close to the one deposited using conventional MAO deposition. At the same time, the influence of the US did not result in a difference in the average size of structural elements. The average diameter of the spheres for all deposited coatings varies in the range of 16.0–18.2 µm, and the average pore diameter varies in the range of 3.8–4.2 µm (Figure 3b). An increase in internal porosity from 28% to 42% and a decrease in surface porosity from 29 to 12% were attributed to the effect of US influence that resulted in the formation of macropores and areas of distorted spheres and their residues (Figure 3c). From Figure 3c, it could be concluded that the UMAOH mode of deposition is a perspective mode of deposition for drug-carrying purposes as the inner porosity and, hence, the volume where the drug could be stored is significantly increased. At the same time, the surface porosity values for UMAOH coatings are close to the conventional MAO deposition (Figure 3c).

**Figure 4:**
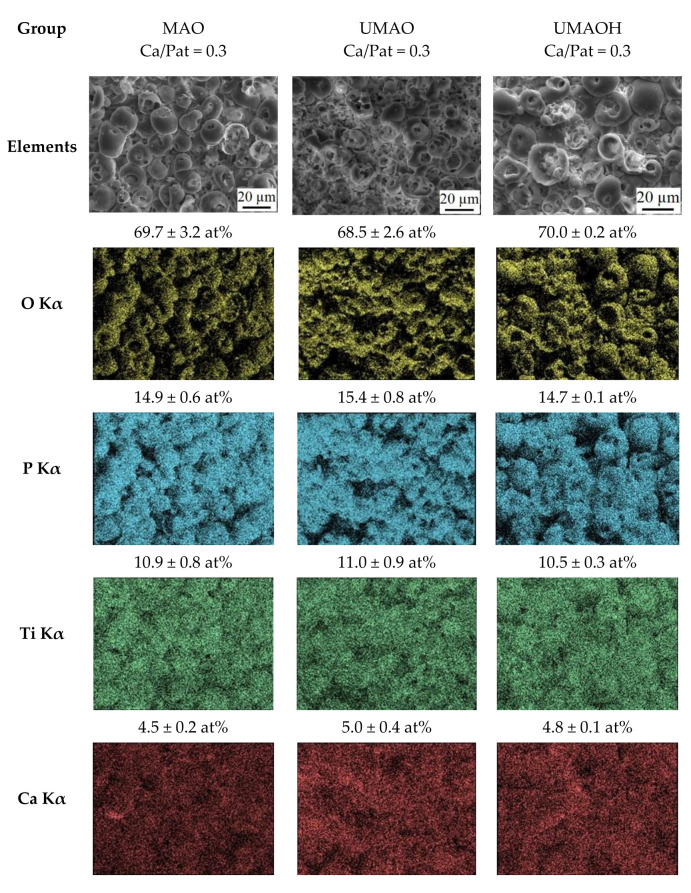
Surface morphology and corresponding EDX mapping of the Ca, P, Ti, and O elemental distribution over the surface of the MAO, UMAO, and UMAOH coatings.

As it is evident from an EDX mapping, there is no significant difference in the elemental composition between all three groups of coatings (Figure 4). The elemental composition of the coatings is as follows: Ca (4.5–5.0 at%); P (14.5–15.5 at%); Ti (10.5–11.5 at%); O (67.5–70.5 at%). The above-mentioned elements are homogeneously distributed through the coatings’ complex morphology. However, the signal shadowing effect during EDX acquisition could lead to a slight distortion of obtained data [47]. It is worth noting that a small amount of calcium present in all types of deposited coatings resulted in a low Ca/P ratio that is equal to 0.3 for all deposited coatings. This has also been reported for conventionally deposited MAO coatings in our previous study [48].

**Figure 5:**
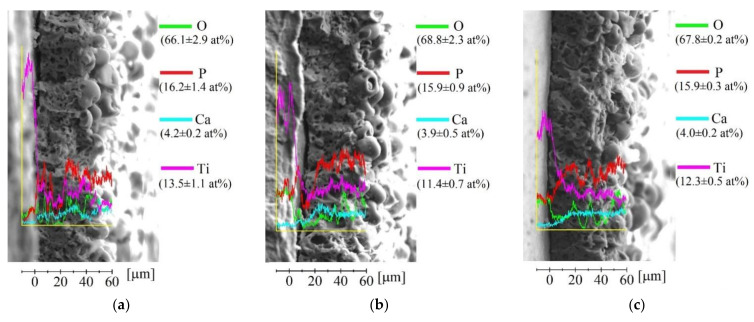
Cross-sectional SEM images and EDX scan lines of the elements (O, P, Ti, Ca) throughout the thickness of the coatings for: (**a**) MAO coating; (**b**) UMAO coating; (**c**) UMAOH coating.

SEM images of coatings’ cross-sections, together with the line distribution of the elemental composition from the substrate to the coating’s surface, are represented in Figure 5. It can be seen from Figure 5 that an elemental distribution in the coatings is characterized by intense fluctuations. This could be due to the elemental localization-associated fluctuations or simply due to the hierarchical porous structure. Nevertheless, the general trends in elemental distribution for deposited coatings are evident. The concentration of titanium decreases while the phosphorous and calcium increases in the direction from the substrate to the top surface of the coating.

**Figure 6:**
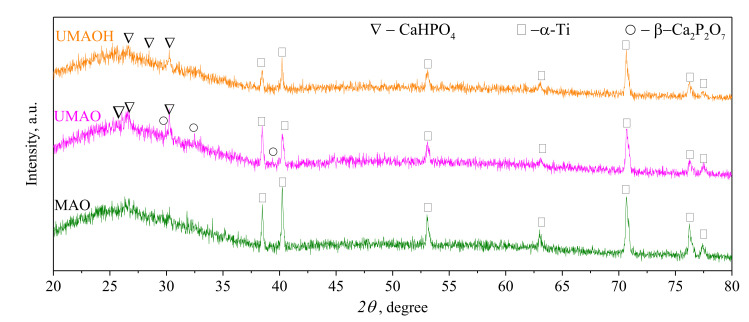
XRD patterns of the MAO, UMAO, and UMAOH coatings. The detected crystalline phases are labeled with corresponding symbols.

XRD results revealed an X-ray amorphous nature of all the MAO coatings, which is confirmed by a pronounced diffusive halo in the range of 2*θ* = 20–35°, represented in Figure 6. According to [49,50], different calcium orthophosphates, including amorphous CaP, are situated in the range of 2*θ* = 20–35°. The signal of high intensity is registered for the α-Ti phase (ICDD No. 00-044-1294), which corresponds to the material of the substrate. For both the US-assisted MAO coatings, in contrast to the control MAO coating, the phase transformation from an amorphous state to an amorphous-crystalline state was observed. In the XRD patterns of UMAO and UMAOH coatings, alongside the diffusive halo arising from the amorphous CaP phase, low-intensive reflexes from monetite (CaHPO_4_, ICDD No. 000-09-0080) and β-calcium pyrophosphate (β-Ca_2_P_2_O_7_, ICDD No. 000-09-0346) phases were observed. The peak intensity from an α-Ti phase was slightly decreased, which could be due to the increase in coatings’ thickness.

Based on the reported results of structure, pore distribution, and roughness, the coatings deposited using the UMAOH regime were chosen as a prospective type for drug-carrying applications. It has been established that UMAOH coatings are characterized by a sufficient set of morphological and structural characteristics among the three studied groups, such as Ra roughness equal to 3.0–4.0 µm, thickness in the range of 50–55 µm, and a combination of high values of internal and surface porosity, which are equal to 39 and 28%, respectively.

### 3.2. Drugs Immobilization and Desorption Kinetics 

**Figure 7:**
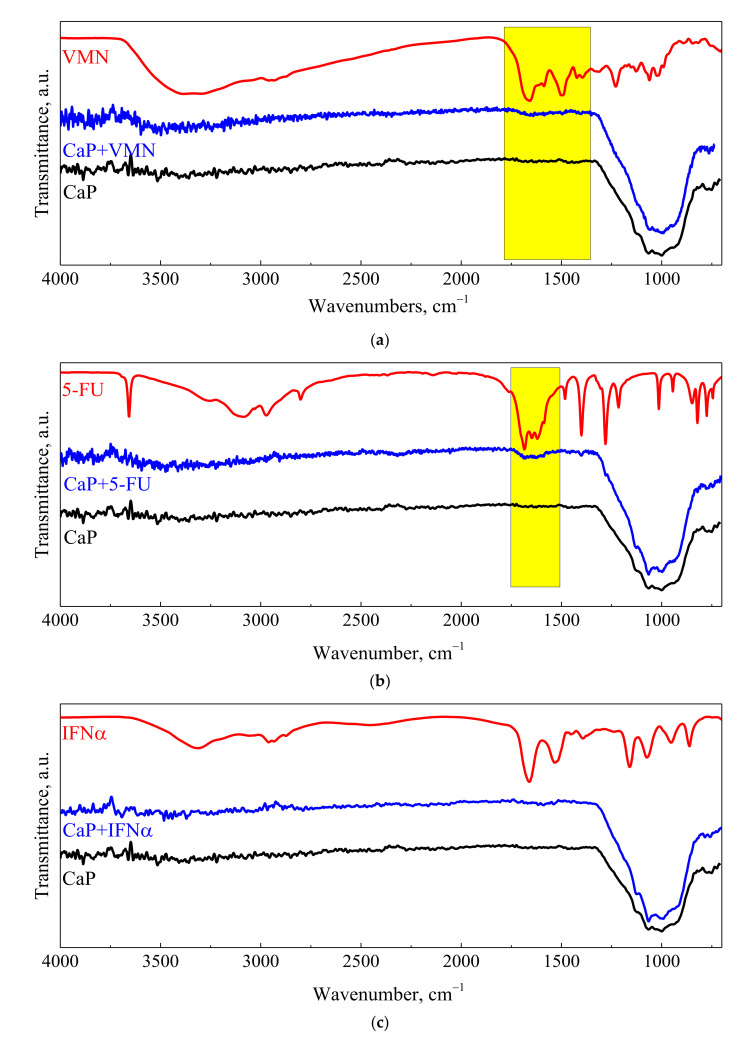
FT-IR spectra of a drug, UMAOH CaP drug-loaded coating, and as-deposited UMAOH CaP coating for the cases of VMN (**a**), 5-FU (**b**), and IFNα (**c**), correspondingly. The region where a signal detected from the VMN or 5-FU drug is observed is highlighted in the yellow box.

After drug immobilization by forced impregnation, UMAOH CaP coatings were characterized using FT-IR spectroscopy. FT-IR spectra of as-deposited UMAOH CaP coating and those loaded with VMN, 5-FU, and IFN drugs are represented in Figure 7. As deposited UMAOH CaP and drug-loaded UMAOH CaP coatings show bonds ascribed to PO_4_^−3^ ions, which are proved by an absorption band region in the range of 1031–1140 cm^−1^. This is in accordance with the published data for an amorphous CaP and a crystalline monetite phase [51].

It is evident that the signals from VMN and 5-FU drugs are superimposed with the signal from as-deposited UMAOH coatings, which are highlighted in the yellow box in the spectra depicted in Figure 7a,b. There is a noticeable absorption in bonds related to C=O stretching at 1658 cm^−1^ for VMN and 5-FU loaded UMAOH CaP (Figure 7a,b). This is a well-reported indication for drug adsorption that has been discussed multiple times in the literature [52,53]. However, more information on the drug adsorption mechanism to the UMAOH CaP surface is hidden due to the large absorption area, in the range of 1300–840 cm^−1^, which could be ascribed to the stretching mode of the PO and P–OH groups (ν PO: 1023, ν P–OH: 914 and 874 cm^−1^) and related to the monetite or β-calcium pyrophosphate phase [54]. Moreover, drug-loaded UMAOH CaP coatings supposedly are carrying a small amount of the drug on their surface as the VMN, 5-FU, or IFNα are soaked into the coatings pore volume upon impregnation. Due to that, in the case of IFNα -loaded UMAOH CaP coatings, there are no distinct lines that could be attributed to the IFNα adsorption alone.

**Figure 8:**
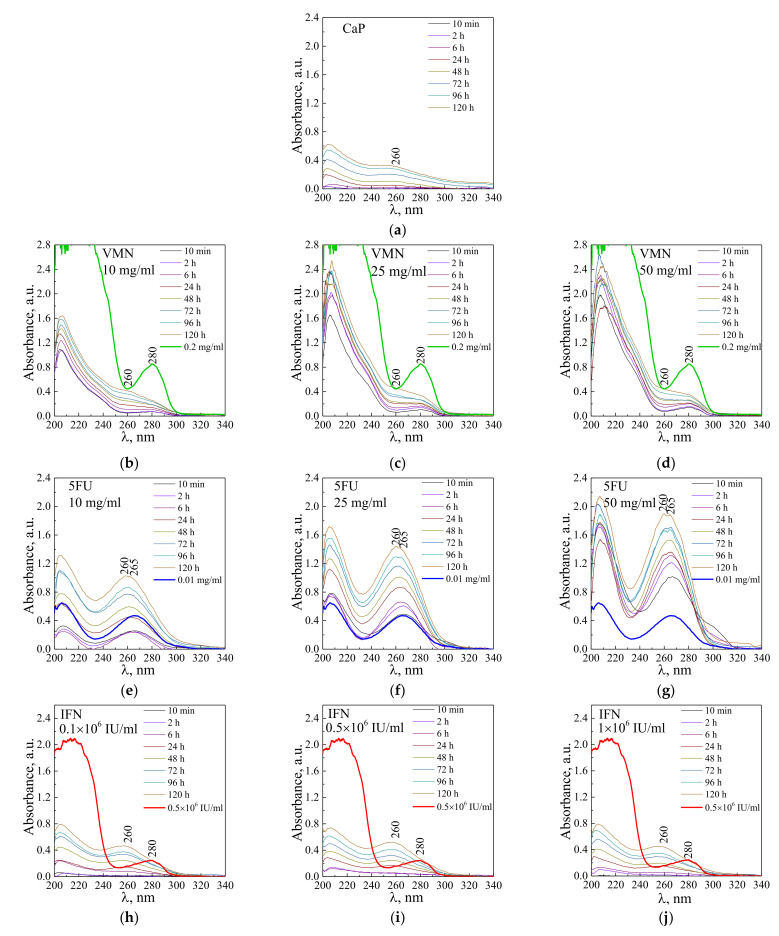
UV−vis absorption spectra of solutions obtained from the as-deposited UMAOH CaP coating (**a**) and UMAOH CaP coating loaded with VMN (**b**–**d**), 5-FU (**e**–**g**), or IFNα (**h**–**j**) for different timepoints. Concentrations of the drug solutions used for loading are as follows: (**b**,**e**) 10 mg/mL; (**c**,**f**) 25 mg/mL; (**d**,**g**) 50 mg/mL. For IFNα concentrations are as follows: (**h**) 0.1 × 10^6^ IU/mL; (**i**) 0.5 × 10^6^ IU/mL; (**j**) 1 × 10^6^ IU/mL.

In Figure 8, UV−vis absorption spectra of solution of VMN, 5-FU, and IFNα released from UMAOH CaP drug-loaded samples for different time exposures are shown. The VMN spectra (Figure 8b–d) show the following trends. With an increase in the concentrations of drug solutions in which the samples were impregnated, the intensity of the absorption bands at a wavelength of 280 nm at the initial moment of drug release increases. The intensity of the absorption bands in the region of 205–210 nm also increases, where VMN is characterized by a high absorption intensity. At the same time, it should be noted that the amount of VMN released did not exceed 0.2 mg/mL for the entire time (Figure 8b–d, green line). With an increase in drug release time, a shoulder having a maximum in the region of 260 nm, which is attributed to a degradation product produced by CaP coating, leads to a smoothing of the peak at a wavelength of 280 nm is evident. The UV−vis absorption peak at 260 nm has been reported for the CaP nanoparticles and antisense oligodeoxynucleotide drug, indicating successful loading in CaP structure [55].

Similarly, when a 5-FU drug gets desorbed from the UMAOH CaP coatings, the intensity of absorption at a wavelength of 265 nm also depended on the amount of drug-loaded (Figure 8e–g). After 24 h of 5-FU release, similarly to previously described VMN results, there is a shift of the maximum at a wavelength of 260 nm for a sample soaked in a solution with a concentration of 5-FU 10 mg/mL (Figure 8e) and a split into two main peaks, being dislocated at 260 and 265 nm for samples soaked in 25 and 50 mg/mL 5-FU solutions, correspondingly (Figure 8f,g).

The obtained spectra indicating IFNα release at the initial time (up to 2 h) are characterized by a weak absorption (Figure 8h–j). The IFNα absorption band with a maximum at 280 nm is, however, below the detection limit (10,000 IU). After keeping the samples in solution for a prolonged time (more than 6 h), a spectrum is formed with gradually increasing absorption bands at a wavelength of 205 and 260 nm. These spectra are similar to those obtained from as-deposited drug-free UMAOH CaP samples, which are probably related to the CaP degradation products (Figure 8a).

**Figure 9:**
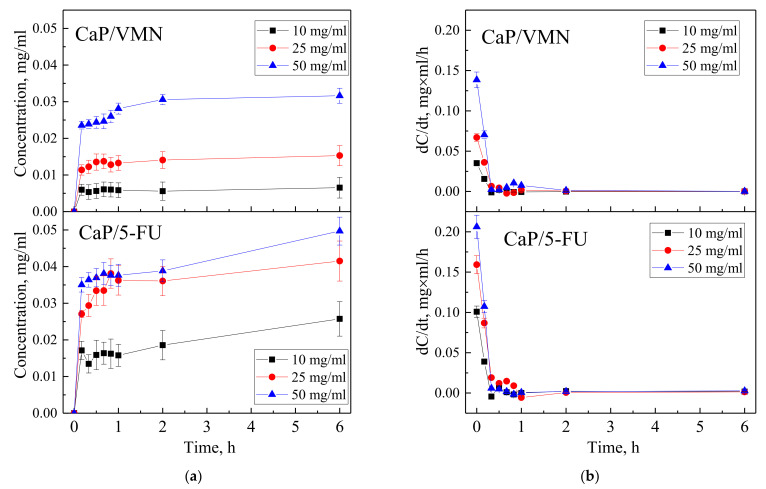
Kinetic curves of desorption (**a**) and release rates (**b**) of VMN and 5-FU drugs that have been loaded into the UMAOH CaP coatings at different concentrations.

Time-dependent curves of VMN and 5-FU drugs released from UMAOH CaP coatings immersed in 0.9% NaCl solution vs. time are shown in Figure 9a. Kinetic curves are characterized by a sharp release of drugs, a so-called initial burst, after which a sustained release begins. After 2 h, the concentration of VMN for samples impregnated by immersion with drug concentrations of 10, 25, and 50 mg/mL is 0.006, 0.014, and 0.031 mg/mL, respectively; after 6 h, it is 0.007, 0.015 and 0.032 mg/mL, respectively. The concentration of 5-FU in solution after 2 h of exposure resulted in the following: The samples impregnated by immersion with drug concentrations of 10, 25, and 50 mg/mL resulted in release of 0.019, 0.036, and 0.039 mg/mL, respectively, and, after 6 h, a release of 0.026, 0.042, and 0.050 mg/mL, respectively. It should be noted that the kinetic curves were plotted based on the results of UV−vis studies (Figure 3), which recorded an additional effect of CaP coating degradation products and modulated the intensity of the signal that is characteristic for the studied drugs after 6 h of exposure. Dependences of drug release rate with exposure time, obtained by differentiating the kinetic desorption curves, showed that for samples impregnated with VMN or 5-FU at various concentrations, the maximum drug desorption rate was reached after 15 min (Figure 9b). At the same time, with an increase in the drug concentration in the initial impregnation solution, the drug release rate increased, which may be the result of a larger volume of drug loaded into the UMAOH coating.

**Figure 10:**
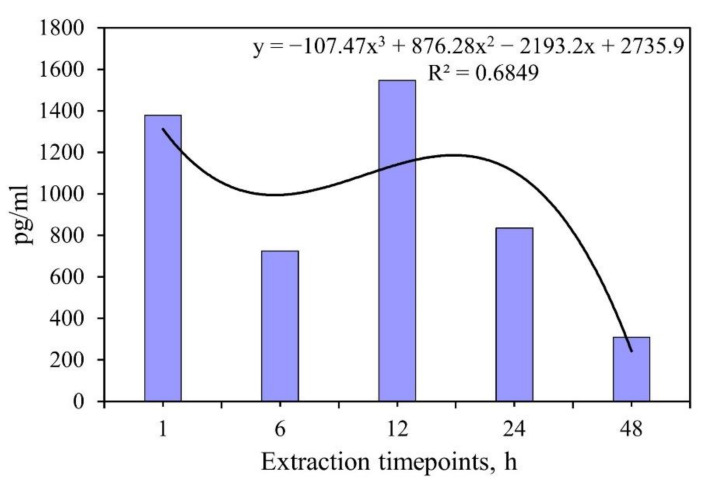
Kinetic curve of release of IFNα from the UMAOH coating in vitro at different time points after immersion in 0.9% NaCl solution. The measurement results are presented for three samples per point.

ELISA showed (Table 1, Figure 10) that a biphasic extraction of IFNα from UMAOH CaP coatings loaded by the drug was observed for 1–48 h. A significant amount of IFNα (up to 13,790–15,460 pg) was released into the solvent after 1 and 12 h of study in vitro. By hour 48 of the immersion study, the median protein content in the solution decreased dramatically, to 3100 pg. Based on the fact that UMAOH CaP coatings’ saturation by IFNα was reached when the samples were immersed in a protein solution with an 1 × 10^6^ ME/mL (4.632 µg/mL of IFNα based on 23.16 µg of IFNα with an 5 × 10^6^ ME/mL activity per ampule, see 2.3.1 item), the coatings absorbed 22 µL of the protein solution, which is equivalent to content of 101,904 pg per sample; the release of IFNα is presented in Table 1. IFNα is a drug approved by the FDA for clinical use in cancer cases due to its antiproliferative and immunomodulatory effects. At the same time, the admission of the drug in high doses (2 × 10^6^ ME per 1 m^2^ of body surface three times a week up to 18 months) [56] often leads to systemic toxicity. Therefore, the loading of IFNα in the UMAOH CaP coating allows two main problems to be solved, mainly, a localized delivery of optimal concentrations of pharmacological agents while, at the same time, maintaining the biological activity of the molecules and reduced systemic toxicity.

The obtained results in Table 1 were also plotted in Figure 10 in order to show a rapid attenuation of the release of IFNα from the UMAOH CaP coating (four to five times by the 48 h time point compared with the rates at 1 h or 12 h). This suggests the physical adsorption of the drug from the solution during the drug loading by immersion [57]. Similar results were obtained by Radin et al. that described the rapid in vitro release of VMN from CaP coatings deposited on Ti-6Al-4V substrates [58]. However, there is a certain difference. Most authors note the exponential [58] or linear in vitro release of drugs (e.g., recombinant BMP-2) [59] from CaP-based carriers. In our case, we did not obtain a linear approximation of the results of the release of IFNα (regression coefficient rs = −0.74; *p* > 0.15) due to a significant scatter in the release of the drug at different time points. CaP degradation contributes significantly to the release of adsorbed molecules [60]. There are the absorbable properties and gradual degradation characteristics of calcium salts [61]. In this regard, it can be assumed that the established “wave-like” kinetics of IFNα release are due to the change in the processes of desorption from the surface of the samples and precipitation of biological molecules from the solution onto the UMAOH CaP coating, as was previously described for calcium ions and inorganic phosphorus during its in vitro degradation. In this regard, monetite particles released from the UMAOH CaP coating into the solution during its dissolution may also be of importance [62]. For example, the size and geometry of CaP submicron particles, including monetite, are important for the in vitro drug release profile [63].

Previously, we have shown the ability of IFNα loaded into copolymer biodegradable capsules to suppress in vitro growth of a culture of Jurkat target leukemia cells [64]. In this regard, the study of the antitumor activity of the UMAOH CaP coating loaded with IFNα was of major interest.

### 3.3. Cytotoxicity Test In Vitro

The MTT assay is a ubiquitous tool in estimating the mitochondrial/metabolic activity, viability, and drug cytotoxicity of eukaryotic cell lines [65], including an estimation of in vitro biocompatibility of biomaterials [66]. Formazan granules (a product of cellular uptake and enzyme-mediated MTT reduction) have been observed in the different intracellular organelles: mitochondria, endoplasmic reticulum, cytosolic lipid droplets, plasma membranes, nucleus, and microsomes [67].

This test has some drawbacks because of its complex interpretation [68]. However, in the case of test drugs (VMN, 5-FU, and IFNα) with inherently anti-metabolic and anti-proliferative effects, MTT is an appropriate tool among other techniques of viability/toxicity evaluation. Furthermore, this technique is applicable in the case of a CaP-based delivery system [63], which allows it to be used in our study.

The results of the MTT-test indicate a weak cytotoxic effect of UMAOH CaP coatings in relation to represented cell lines. The decrease in the viability of different cells varies from 10 to 25% relative to the control. The greatest decrease in viability was observed in the case of the MCF-7 cell line contacted in vitro with UMAOH CaP-coated samples. According to ISO 10993-5-2009, a cytotoxic effect is considered if the reduction in cell viability exceeds 30%. Therefore, notable cytotoxicity of as-deposited UMAOH CaP coating against either non-tumorous (3T3) or tumorous cell lines (HeLa, MCF-7) was not observed.

However, the observed effect requires some explanation. We assume that the products of the soluble UMAOH CaP coating, primarily calcium ions [62], are involved in the inhibition of cell growth. Herein, an increasein extracellular Ca^2+^ level briefly provokes free intracellular (Ca^2+^) augmentation [69], resulting firstly in the calcium-mediated cell death of cancer cells, including the MCF-7 line [70].

**Figure 11:**
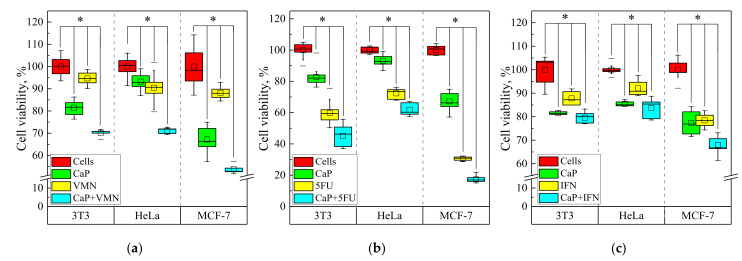
Results of MTT estimation of in vitro viability of 3T3, HeLa, and MCF-7 cell lines when incubated with as-deposited UMAOH CaP coatings and loaded with VMN (**a**), 5-FU (**b**), and IFN (**c**). The data are presented as a box plot. (* statistically significant differences at *p* < 0.005 compared to the control group (cells) according to the Mann–Whitney U test).

UMAOH coatings loaded with VMN show a weak cytotoxic influence on the 3T3 and HeLa cell lines but, to a greater extent, affect the viability of the MCF-7 cell line; the number of viable cells was 70, 71, and 54%, respectively. At the same time, the antibiotic alone does not have a pronounced cytotoxic effect, even at a concentration of 0.06 mg/mL (Figure 11a).

In turn, CaP coatings that contained cytostatic anti-metabolic drug 5-FU have a significant cytotoxic effect in all studied cell lines (Figure 11b). This antineoplastic agent possesses nonspecific side toxic effects in healthy cells [71]. Therefore, decreased viability of both cancer HeLa cells (~70 % of living cells) and healthy 3T3 fibroblasts (~60 % of viable cells) treated with 5-FU alone occurred. A CaP based drug delivery system statistically elevated 5-FU-mediated cytotoxicity (Figure 11b). Especially, the viability strongly diminished, from 30% in the case of a 5-FU drug control, to 20 % in the case of UMAOH CaP loaded with 5-FU in the case of the MCF-7 cancer cell line (Figure 11b). MCF-7 cell sensibility to 5-FU is well-known [72]. Due to the available data, we can conclude that the UMAOH CaP coatings that were loaded with VMN of 5-FU enhanced cell damages caused in all cell lines (Figure 11a,b).

Furthermore, the IFNα drug caused in vitro cell death of healthy 3T3 cells, as well as HeLa and, especially, MCF-7 cancer lines. Cell viabilities were significantly reduced (in the range of 10–20%) compared to 100% of cells in the control group (Figure 11c). IFNα-induced inhibition of cell growth of 3T3 [73], HeLa [74], and MCF-7 cells [75] is well documented. UMAOH CaP coating loaded with IFNα induced only intensive mortality of MCF-7 target cells compared to other used cell lines in vitro (up to 30% of non-viable cells). There were statistical differences detected compared with both control groups: as-deposited UMAOH coating and IFNα drug (Figure 11c).

Breast cancer cell line MCF-7 is susceptible to IFNγ [76] and IFNβ [77]. In turn, IFNα, even at low concentrations (500 IU/mL), exhibits in vitro cytotoxicity against MCF-7 tumor cells through increased autophagy processes. In addition, IFNα enhances the senescence-related phenotype of tumor cells to other apoptotic stimuli [72,78]. MCF-7 cells are sensitive to an increase in intracellular Ca^2+^ content, which induces their apoptosis [70] in response to an increase in Ca^2+^ in the extracellular space [79]. In this regard, IFNα impregnated in soluble UMAOH CaP coating can potentiate apoptotic stimuli of Ca^2+^ ions released during the biodegradation of such a drug delivery system.

### 3.4. Antibacterial Studies

The disk diffusion method on agar medium is the most used technique for antibiotic susceptibility testing, applied also for *S. aureus* in vitro growth [80]. The technique is based on inhibition zone diameter measurement around the corresponding disk containing the antibiotic. Such a technique is also applicable in the case of a CaP-based delivery system carrying various types of antibiotics [63]. That fact allowed us to use this technique in our research. As is known from the literature, *S. aureus* is the bacterium most frequently detected in preimplant infections [66].

**Figure 12:**
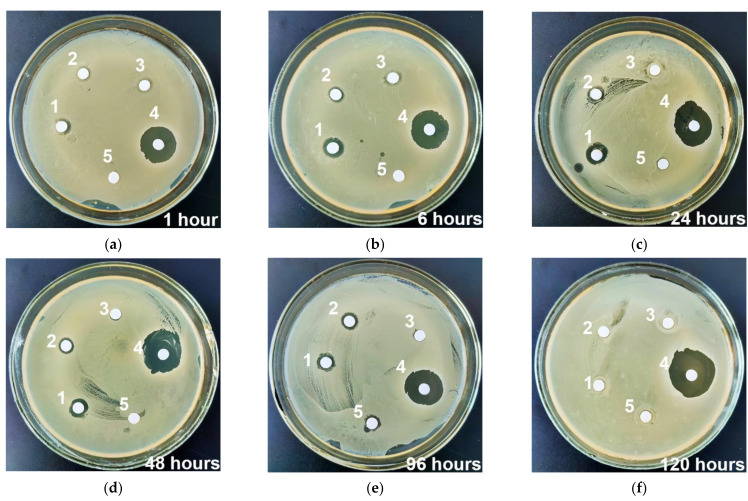
Inhibition zone of 24-h extracts of UMAOH CaP coatings against methicillin resistant *S. aureus* for different time points: (**a**) 1 h; (**b**) 6 h; (**c**) 24 h; (**d**) 48 h; (**e**) 96 h; (**f**) 120 h. The numbers depicted in the image correspond to extracts of the coatings immersed in VMN of (1) 50 mg/mL concentration, (2) 25 mg/mL concentration, and (3) 50 mg/mL concentration. Positive control of bactericidal action (4) VMN of 20 µg/mL concentration; Negative control of bactericidal action (5) an extract of as-deposited UMAOH CaP coating.

Extracts obtained from VMN-loaded UMAOH CaP coatings already begin to show antimicrobial activity after one hour of immersion (Figure 12). Table 2 shows the dimensions of the width of the zone of inhibition of bacterial growth in solutions saturated with VMN extracted from samples. An increase in the exposure time of VMN-loaded UMAOH CaP coatings in solutions having concentrations of 10, 25, and 50 mg/mL shows a dose-dependent (by Wilcoxon test) increase in the bacterial inhibition zone H up to 24 h, followed by a monotonous decrease in bactericidal activity until 120 h of incubation. It should be noted that the obtained results are in good agreement with the quantitative assessment of the release of VMN from the samples (Figure 10) and with the results of measuring the UV-Vis spectra of solutions (Figure 9). As deposited, UMAOH CaP coatings show a minor antibacterial effect at 96 h.

The inhibition zone observed in the experiment is well correlated to the already published studies of antibacterial activity of VMN [81]. Thus, the extracts of UMAOH CaP carriers with VMN can inhibit bacteria growth more effectively in vitro as compared to pure VMN drug (Table 2, Figure 12). A similar potentiating effect of calcium salts in VMN-loaded calcium-based carriers has been found to inhibit the growth of *S. aureus* [61] and methicillin-resistant *S. aureus* [82].

The observed effect of an existing inhibition zone overgrowth for all samples kept in NaCl solution for up to 7 days may be due to the adaptation of MRSA to antimicrobial components, released due to the degradation of the CaP coating. In the paper by L. Polo et al. [83] and V. Uskokovic et al. [84], an antimicrobial activity of CaP has already been observed, however, the mechanism of action was not described. Despite the biodegradation of CaP based coatings, it has been documented that such material has high affinity to *S. aureus* [85]. Therefore, further research is needed for understanding the intrinsic antibacterial activity of CaP-based coatings and the potential synergy between the coating and the antibiotics loaded in its volume.

## 4. Conclusions

In the present study, we described a way to manufacture a drug delivery system based on CaP coating. Characterization of CaP coatings resulted in the fact that, to produce a material suitable for drug-carrying applications, a hybrid regime of US field should be used in the course of the UMAOH process. In that way, the optimal type of coating in terms of its physicomechanical properties could be formed. The coatings that have the following parameters: Ra roughness equals 3.5 µm, thickness in the range of 50–55 µm, and a combination of high values of internal and surface porosity which are equal to 39 and 28%, respectively, were used for drug loading. Drug loading into the UMAOH CaP coatings was confirmed by FT-IR, UV−vis desorption kinetics, and ELISA, in the case of the IFNα drug. Kinetic curves were characterized by an initial burst, after which a sustained release of VMN or 5-FU begins. A “wave-like” kinetics of IFNα release has been established during the study and is associated with the change in the processes of desorption from the surface of the samples and precipitation of biological molecules from the solution onto the UMAOH CaP. The cytotoxic effect of as-deposited UMAOH CaP coating was not observed for 3T3, HeLa, and MCF-7 cell lines. 

However, it was established that the UMAOH CaP coatings loaded with VMN or 5-FU enhanced cell death in all healthy and tumor cell lines. The reported findings provide a perspective for the development of anticancer drug delivery systems to suppress tumour growth (5-FU) and reduce the risk of infectious complications (VMN) during malignant progression and/or its cytostatic treatment. 7Seventy percent survival of healthy 3T3 cells caused by UMAOH CaP coating loaded with VMN is the boundary value of in vitro cytotoxicity, according to the standard ISO10993-5. However, the antibacterial activity of drug delivery systems, such as those presented in this work, may be useful in implants that are made for osteosynthesis of bone fractures complicated by infection.

It was also established that IFNα impregnated in soluble UMAOH CaP coating can potentiate apoptotic stimuli during the biodegradation, which also could be of benefit. The antibacterial activity of VMN-loaded UMAOH CaP coatings reached its peak at 24 h of incubation, after which a monotonous decrease in activity was observed. Thus, we described an approach to manufacture a drug delivery system based on CaP coating that could carry several types of different drugs and modulate their mechanisms of action.

## Figures and Tables

**Table 1:** IFNα in vitro releasing (% of initial level) from UMAOH CaP coatings loaded by protein, at the different immersion times, Me(Q1–Q2).

Extraction Time, h	IFNα Amounts in Extracts, pg and % of Release Compared with Initial Content of 101,904 pg per Sample
1	13,790 (12,750–14,850) 13.5 (12.5–14.6)%
6	7250 (6100–8280) 7.1 (6.0–8.1)%
12	15,460 (14,960–15,950) 15.2 (14.7–15.7)%
24	8350 (6440–16,670) 8.2 (6.3–16.4)%
48	3100 (2750–3490) 3.0 (2.7–3.4)%

**Table 2:** Inhibition zone caused by extracts of UMAOH CaP coatings prepared at the different immersion times against methicillin-resistant *S. aureus* for 24h of incubation.

No.	Sample Group	Width of t *S. aureus* Inhibition Zone (*H*) for Different Time Points, mm					
	*n* = 3	1 h	6 h	24 h	48 h	96 h	120 h
1	UMAOHCaP (VMN 50 mg/mL)	0.9 (0–1.1) ^a,b^	1.8 (1.4–2) ^a,b^	3.2 (2.8–3.3) ^a,b^	2.1 (2.1–2.7) ^a,b^	1 (1–1.3) ^a,b^	0 ^b^
2	UMAOHCaP (VMN 25 mg/mL)	0.3 (0.1–1) ^a,b^	1 (0.9–1.1) ^a,b^	1.5 (1.4–2) ^a,b^	1 (0.1–1.2) ^a,b^	0.8 (0.3–1) ^b^	0 ^b^
p_T__1_ < 0.05							
3	UMAOHCaP (VMN 10 mg/mL)	0.1 (0–0.1)^a,b^	0.3 (0.3–0.9) ^a,b^	0.1 (0.1–0.1) ^b^	0.1 (0–0.2)^b^	0.2 (0.1–0.3) ^b^	0 ^b^
p_T__1,2_ < 0.05							
4	VMN 20 µg/mL (Positive control of bactericidal action)	7.2 (7.2–8.9)	9.2 (7.1–9.5)	9.3 (8.6–9.8)	8.4 (8–8.5)	8.4 (7.4–8.9)	8.6 (7.8–8.6)
5	UMAOH CaP (Negative control of bactericidal action)	0 ^b^	0 ^b^	0 (0–0.1) ^b^	0 (0–0.4) ^b^	0.4 (0.1–0.7) ^b^	0^b^

Note: n—number of Petri dishes studied in each group; ^a^ statistical difference with a negative control group; ^b^ statistical differences with a positive control group according to Mann–Whitney U-test. p_T__n_—statistical difference with corresponding group according to Wilcoxon-test.
